# Allele frequency and gene expression differences under key winter stresses in temporal populations of two timothy cultivars

**DOI:** 10.1007/s00122-026-05270-1

**Published:** 2026-05-23

**Authors:** Akhil Reddy Pashapu, Sigridur Dalmannsdottir, Marit Jørgensen, Mallikarjuna Rao Kovi, Odd Arne Rognli

**Affiliations:** 1 Department of Plant Sciences, Faculty of Biosciences, Norwegian University of Life Sciences, PO Box 5003, 1432 Ås, Norway; 2 Department of Grassland and Livestock, Norwegian Institute of Bioeconomy Research, 9016 Holt, Tromsø, Norway

## Abstract

Timothy is the most important perennial forage grass species in northern Norway, a region that is predicted to experience variable winter weather conditions due to climate change. Knowledge about how timothy cultivars respond to a changing climate is crucial for safeguarding forage production at higher latitudes. In the current study, we investigated changes in gene expression under freezing and ice encasement stresses and SNP allele frequencies between temporal populations (seed generations) of the two northern-adapted timothy cultivars Engmo and Noreng. In general, there was a decrease in freezing tolerance (defined as LT_50_, the temperature lethal to 50% of the population) and an increase in ice encasement tolerance (defined as LD_50_, the duration lethal to 50% of the population) over time. Comparative transcriptome analyses identified several genes known to be involved in stress responses, such as ethylene-responsive transcription factors, dehydration-responsive element binding transcription factors, reversion to ethylene sensitivity 1, and abscisic acid repressor 1, as differentially expressed between the temporal populations of Noreng under freezing stress. Several loci with large allele frequency changes were observed to be in close proximity to the genes displaying patterns resembling shifts over time in Noreng. Very few gene expression differences between populations of both cultivars under ice encasement stress could be due to weak selection pressure during seed multiplication. There was a gradual decline in genetic diversity in populations of both cultivars over time. The results indicate that phytohormone-mediated transcriptional regulation might be one of the key mechanisms for adaptation to changing winter weather conditions at higher latitudes. These findings underscore the importance of monitoring genetic shifts during seed multiplication to maintain cultivar stability and suggest that the identified stress-responsive genes could serve as valuable targets for breeding climate-resilient forage crops.

**Supplementary Information:**

The online version contains supplementary material available at 10.1007/s00122-026-05270-1.

## Introduction

Climate change is expected to cause more pronounced changes in temperature and precipitation patterns at higher northern latitudes than elsewhere, with greater variability and increase in temperatures during winter than in summer (Overland et al. 2011; IPCC 2014; Post et al. 2019). The agricultural land at these northern latitudes is predominantly used for the cultivation of perennial forage grasses. In Northern Norway, future climate changes are expected to extend the growing season by 1–4 weeks in the period 2021–2050 relative to 1961–1990 (Uleberg et al. 2014). The development of plant material that is sufficiently robust and at the same time able to make use of a longer growing season is therefore an important strategy for adaptation to climate change. Though in general, the persistence and stable forage yield of these grasses are primarily determined by their ability to survive harsh winter conditions, the increase in the number of cuts per growing season in recent years, due to relatively longer growing seasons, is also suspected to affect the persistence of the timothy. Winter survival is a complex trait determined by several winter stresses in the field, including tolerance to freezing temperatures, long-term ice encasement, waterlogging, winter desiccation, and snow mold infestation (Sandve et al. 2011; Rognli 2013). The increase in winter temperatures is likely to have a negative impact on winter survival of perennial forage grasses due to poor cold acclimation, reduced snow cover, and frequent ice encasement events (Rapacz et al. 2014; Dalmannsdottir et al. 2017; Jørgensen et al. 2020). To ensure their survival under a changing climate, perennial grasses at higher latitudes must respond simultaneously to greater variability within a winter season and an increase in mean winter temperatures over the years.

All living organisms must adapt and evolve to the changes in the local environment or evade unfavorable conditions to ensure their survival and successful reproduction. The ability of any given plant species to avoid extinction and persist under rapidly changing climatic conditions depends on the rate of migration (range shift), the extent of plasticity, and the speed of adaptive evolution (Nicotra et al. 2010; Chen et al. 2011; Hoffmann and Sgrò, 2011; Anderson et al. 2012b). While migration (range shift) and plasticity offer short-term survival by evading or buffering against unfavorable conditions (Davis and Shaw 2001; Lenoir et al. 2008; Anderson et al. 2012a; Stollewerk et al. 2025), they are often insufficient or may even hinder long-term adaptation (DeWitt et al. 1998; Cunze et al. 2013; Oostra et al. 2018). Therefore, the long-term persistence of a species is ultimately dependent on its capacity to evolve in response to macro climatic trends (Aitken et al. 2008; Chevin et al. 2010; Franks et al. 2014; Becklin et al. 2016; Mitchell and Whitney 2018). Understanding the evolutionary responses of plant species under a changing climate is crucial for safeguarding sustainable forage production at higher northern latitudes (Thorsen and Höglind 2010; Hart et al. 2022; Thivierge et al. 2023). This knowledge would be useful for breeding resilient plant varieties with stable yields under variable weather conditions, and/or to identify and prioritize the conservation practices for species at risk of extinction to mitigate biodiversity loss.

Studying genetic differentiation between ancestor and descendant populations is a powerful approach to identify contemporary evolution in natural populations (Franks et al. 2008, 2018). Recently, some studies have resurrected the seeds from the past and compared their phenotypes, physiology, gene expression, and allele frequencies to their contemporary descendant populations (Franks 2011; Nevo et al. 2012; Franks et al. 2016; Rauschkolb et al. 2022). These studies provide evidence that natural plant populations can undergo rapid evolution in response to changing climate conditions.

As of 2024, two-thirds of the total agricultural land in Norway is under leys, meadows, or permanent grasslands (Statistisk sentralbyrå, 2025), signifying the importance of grasslands in Norwegian agriculture. Among the forage grasses, timothy (*Phleum pratense* L*.*) (2n = 6*x* = 42) is by far the most widely cultivated forage grass in Norway (Havstad and Aamlid 2013; Steinshamn et al. 2016). Several reports from farmers in recent years have indicated increased winter kill and reduced persistence of timothy cultivars in northern Norway (Jørgensen 2017). This can be due to new management regimes (increasing number of cuts/season), genetic shifts due to seed multiplication of the northern timothy cultivars further south, and climate change. The synthetic cultivar ‘Noreng’ released in 2002 replaced the old landrace ‘Engmo’ (released in 1953) on the market in Northern Norway, since it yielded as good or slightly better than ‘Engmo’ in official variety trials with more yield in the second cut and comparable winter survival. However, based on the reports from farmers, we suspected that the synthetic cultivar Noreng might have experienced genetic shifts over time since it was created.

The main objective of the present study was to investigate differences in allele frequencies and survival under key winter stresses by resurrecting three temporal populations of the timothy cultivars Engmo and Noreng. We aim to achieve the objective by studying (1) survival and gene expression differences under major winter stresses, i.e., freezing stress and ice encasement stress, (2) changes in allele frequencies and genetic differentiation between the temporal populations. The idea is that when the temporally separated populations, the ancestor and its descendant populations, were subjected to similar stress conditions, the gene expression differences between populations could be due to plasticity or adaptation, and the non-random changes in allele frequencies and gene expression could be indicative of adaptive responses.

## Materials and methods

### Plant material

The timothy cultivars used in the current study are Engmo (Eng), an old very winter hardy landrace originating from a farm in Troms County (68.93719 N, 18.05695 E) and released as a cultivar in 1953, and Noreng (Nor), a synthetic cultivar based on ten clones of ‘Engmo’ and four clones of the south-Norwegian timothy landrace ‘Grindstad’ (59.42445 N, 11.34564 E). The three temporal populations of Engmo were seedlots from the years 1988 (early), 2003 (mid), and 2020 (current), while the three populations from Noreng were from the years 1998 (early), 2010 (mid), and 2020 (current). The early populations were from pre-basic seedlots, while the mid and current populations were obtained from certified seedlots. For the sake of brevity, the populations will be referred to as early, mid, and current from hereon. The seedlot of the Engmo Mid population was obtained from Iceland but originates from seeds produced in Canada, as seedlots from this period produced in Norway were unavailable.

### Freezing tests

The plants raised from the seedlots were initially grown for six weeks at 18 °C and 24 h light. Later, these six-week-old plants were cold acclimated initially for 2 weeks at 9 °C, followed by 1 week at 6 °C, and finally for two weeks at 2 °C under 12 h light (55 PPFD). After cold acclimation, the plants were trimmed to 4 cm (3 cm shoot and 1 cm roots) and placed in plastic boxes with humid fine sand and temperature loggers. Thereafter, the boxes were transferred to the freezers. Temperature loggers were put in each box. To prevent supercooling, the temperature was lowered from 2 °C to − 3 °C by 1 °C h^−1^ and the plants were kept at − 3 °C for ~ 12 h followed by lowering by 1 °C h^−1^ from − 3 °C to − 10 °C and by 3 °C h^−1^ until the pre-set temperatures (− 12, − 15, − 18, − 21, − 24, − 27 °C) were reached. At these temperatures, a subset of boxes was removed from the freezers and placed at 2 °C in the dark for thawing overnight. A set of replicates was kept unfrozen at 2 °C in the dark for control. Two replicates of eight plants each were used per entry at a given freezing temperature for testing the freezing tolerance and regrowth. For transcriptomic studies, two replicates of two plants for each entry were placed in separate boxes, which were withdrawn from the freezers when the pre-set temperature was reached. After thawing, the plants were transplanted into trays and scored for survival after four weeks of regrowth in growth chambers at 18 °C, 24 h light (ca. 150 PPFD). The median lethal temperature (LT_50_) was estimated using a generalized linear model (glm) with a binomial distribution separately for each cultivar. Temperature and population, and their interaction, were modeled as fixed effects. The *emmeans* function from the *R* package emmeans (Lenth 2017) was used to estimate marginal means of survival probability at − 21 °C for all populations. Pairwise comparisons between populations of each cultivar were performed using Tukey’s HSD method via the pairs() function of *emmeans* to determine if there were significant differences in survival rates at *p* < 0.05. For the transcriptomic study, we decided to use the plant material from − 18 (< LT_50_) and − 24 (> LT_50_) temperatures as *T*1 and *T*2, respectively, while cold-acclimated (CA) plants were used as controls. The rationale was to capture the gene expression differences between populations and cultivars below and above LT_50_. Due to limited biological replicates (two replicates with two plants each), we chose to perform two RNA extractions (one from each plant) from one of the replicates, while the two plants from the other replicate were pooled and extracted as one. RNA extractions were carried out using Qiagen RNeasy Plant mini kit as per manufacturer guidelines. After inspecting the purity and integrity of the RNA extractions, the samples were shipped to Novogene Co Ltd (Cambridge, UK), where the paired-end sequencing at 150 base pair read length (~ 20 million raw reads/sample) was performed using Illumina NovaSeq 6000 platform.

### Ice encasement tests

Plants for ice encasement tests were prepared and acclimated in the same manner as described in freezing tests. The cold-acclimated plants were placed into boxes, covered with ice, followed by adding ice water to fill the gaps. The boxes were covered (not sealed) with a lid and kept at − 2 °C in the dark. A subset of boxes was removed from the freezing room after 22, 36, 54, 68, 75, and 82 days. After thawing the boxes at room temperature, the plants were then transplanted into trays, similarly to the plants in the freezing test, and scored for survival and regrowth. The median lethal number of days (LD_50_) was estimated in the same manner as LT_50_, by using days under ice encasement and populations as fixed effects. Plant material withdrawn at 36 (< LD_50_) and 68 (> LD_50_) days were used as T1 and T2, respectively, and CA plants were used as controls. Differences in survival rates were estimated at 54 days using marginal means. RNA extractions and sequencing were carried out as described above in the freezing tests.

### Plant propagation, DNA extraction, and sequencing

For population genetic studies, approximately 80–100 seeds of each population were sown in two pots and cultivated at ambient room temperatures for 8 weeks. For DNA extraction, approximately an equal amount of tissue from 30 randomly selected plants from each pot was collected and flash-frozen in liquid nitrogen. The collected plant tissue was ground, and DNA extractions were carried out using Promega wizard HMW DNA extraction kit according to the manufacturer’s guidelines. After inspecting the quality and the concentration of the DNA extractions, the DNA was shipped to BMKgene, Germany, for high-throughput sequencing using specific locus amplified fragment sequencing (SLAF-seq) (Sun et al. 2013). Based on the results of in silico pre-design, restriction enzymes *RsaI* and *HaeIII* were chosen for library preparation as they generated a uniform distribution of SLAF tags (400 k) across the genome. All samples were paired-end sequenced using Illumila NovaSeq X platform at 150 base pair read length and an average of 30 × coverage, yielding ~ 16–20 million reads per sample.

### RNAseq read alignment, abundance estimation, and functional annotation

Adapter removal and trimming low-quality bases from the raw reads were carried out using fastp (Chen et al. 2018) with options *length_requir*ed 100, *cut_front*, and *cut_window_size* 4. After inspecting with FastQC (Andrews 2010), the trimmed reads were aligned to the draft genome of timothy (unpublished) using STAR (Dobin et al. 2013) with default options. The resulting BAM files were used to estimate abundance at the gene level using featurecounts from subread (Liao et al. 2014). For functional annotation, the CDS sequences of the genes extracted using gffread (Pertea and Pertea 2020) were blasted against the protein sequences of the *Triticum aestivum* downloaded from NCBI and Ensembl Plants using Diamond (Buchfink et al. 2021) with options*–ultra-sensitive* and e-value cutoff of 1e-6.

### Differential expression analysis of freezing and ice encasement samples

One of the objectives of the study was to identify gene expression differences between temporal populations. Differential expression and all further downstream analyses were carried out in *R* (4.3.3) (R Core Team 2022). Initially, genes with low read count were filtered out by the *filterByExpr* function from the edgeR package (Robinson et al. 2010) with options min.count = 20, and grouping at biological replicates. Principal component analysis (PCA) based on all genes retained after filtering was carried out to identify the main sources of variation. A glm with negative binomial distribution was used to identify DEGs. To identify genes with differential expression between populations of a cultivar, contrasts were performed at respective treatments between all possible combinations using DESeq2 (Love et al. 2014). For example, contrasts were performed between Early_CA vs Mid_CA, Mid_CA vs Current_CA, Early_CA vs Current_CA, and similarly at T1 and T2. Furthermore, contrasts were performed between cultivars of respective populations (Eng_Early_CA vs Nor_Early_CA, Eng_Mid_T1 vs Nor_Mid_T1) to identify genes with differential expression between cultivars. To account for biological noise and reduce false positives, differential expression was tested against a minimum log-fold change threshold ≥ log2(1.2), and only genes with a Benjamini–Hochberg adjusted *p* < 0.05 were considered differentially expressed. Gene ontology (GO) enrichment analyses were performed using clusterProfiler (Wu et al. 2021). The approach mentioned above was used to identify DEGs in both freezing and ice encasement tested samples.

### Population genetic analysis

The quality of the clean reads (adapter removal and trimming for base quality) received from the sequencing service provider was inspected using FastQC (Andrews 2010). After verifying their quality, the reads from biological replicates were concatenated before they were aligned to the draft genome of timothy using bwa mem (Li 2013) with default options, followed by sorting the BAM files by coordinates using samtools (Danecek et al. 2021). The BAM files were processed using grenedalf (v0.5.1) (Czech et al. 2024) with quality filters *–sam-min-base-qual 25, –sam-min-map-qual 20, –filter-sample-min-count 2, –filter-sample-min-read-depth 60, –filter-sample-max-read-depth 500, -filter-total-snp-min-frequency 0.05,* and *–pool-sizes 360* to estimate allele frequencies, genetic diversity. Nucleotide diversity (*θπ*), watterson’s θ (*θw*), Tajima’s *D* and genetic differentiation (*F*_st_) were estimated along 1 kilobase (kb) sliding windows. Only windows with at least 5 valid single nucleotide polymorphisms (SNPs) across all populations were retained. The genome-wide genetic diversity is calculated as the mean of the retained windows. Genetic differentiation (*F*_st_) was estimated using the unbiased Hudson method between all possible pairs (Early vs Mid, Mid vs Current, and Early vs Current). The statistical significance of the *F*_st_ windows is determined as described in Franks et al. (2016). To briefly explain, the test distribution was regressed on a null distribution created by bootstrap resampling with 1000 replicates using the outlierTest function from the package CAR (Fox et al. 2001). The *p* values were corrected for multiple tests using fdrtool (Klaus and Strimmer 2006) with FDR *q*-values < 0.05. Further, the F_st_ windows were filtered based on criteria (1) the window has ≥ 5 SNPs in all three populations of a cultivar and (2) a *F*_st_ > 0.15 in at least two pairwise comparisons between populations of a cultivar. Genes overlapping with the *F*_st_ windows passing the above filters were identified using the function findOverlaps from the *R* package GenomicRanges (Lawrence et al. 2013). For functional annotation, CDS sequences of the genes overlapping with the *F*_st_ windows were blasted against the protein sequences of *Triticum aestivum* as described in the previous section. Absolute allele frequency difference (*|ΔAF|*) was estimated as *|ΔAF|* =*|AF Early – AF Current|*. After selecting SNPs whose AF in Mid population is intermediate to the AF of Early and Current populations, a custom R function was used to find genes displaying shifts near the SNPs among the 95th percentile of *|ΔAF|*.

## Results

### Freezing and ice encasement tolerance in temporal populations

The temporal populations of Engmo and Noreng differed in freezing tolerance (FT) and ice encasement tolerance (ICET), which are measured as LT_50_ and LD_50,_ respectively. There was a gradual decrease in FT and an increase in ICET from early to current populations in Engmo (Table 1 and Figure S1). A similar trend is observed in the Noreng populations, except for FT and ICET of the Mid population (Table 1 and Figure S1). The freezing stress survival (at − 21 °C) was significantly lower in Mid and Current compared to Early populations in Engmo, while there were no significant differences between populations of Noreng. Under ice encasement, only the survival rate (at 54 days) of Mid population was significantly higher than Early population in Noreng (Table 1).

**Table 1:** Freezing tolerance (LT_50_) and ice encasement tolerance (LD_50_) in temporal populations of Engmo and Noreng. Statistical differences were determined using Tukey’s HSD method via the pairs() function of emmeans, with significance assessed at *p* < 0.05. Populations sharing the same superscript do not significantly differ in survival rates at − 21 °C under freezing stress and 54 days under ice encasement

	Freezing tolerance (LT_50_)		Ice encasement tolerance (LD_50_)	
	Engmo	Noreng	Engmo	Noreng
Early	− 22.7^a^	− 21.8^a^	52.9^a^	49.3^a^
Mid	− 20.1^b^	− 21.8^a^	58.0^a^	59.9^b^
Current	− 19.3^b^	− 20.0^a^	59.3^a^	52.8^ab^

### RNAseq read alignment and functional annotation

Trimming reads for base quality and adapters removed ~ 3–4% of reads in all samples. The alignment rate was > 85% for all samples except for the samples which were encased in ice (*T*1 and *T*2). In ice encased samples, the alignment rate decreased with an increase in duration under ice encasement in all populations of both cultivars. Taxonomic classification of clean RNAseq reads of ice encased samples by kraken2 (Wood et al. 2019) using PlusPF (v06/05/2024) revealed that the proportion of reads assigned to bacteria was *T*2 > *T*1 > CA, indicating an increase in relative abundance of bacterial sequences with duration under ice encasement. Homology-based blast search against protein sequences of *Triticum aestivum* downloaded from NCBI and Ensembl Plants annotated 107,945 (92%) and 108,766 (93%) genes, respectively.

### Differential gene expression under freezing stress

**Fig. 1:**
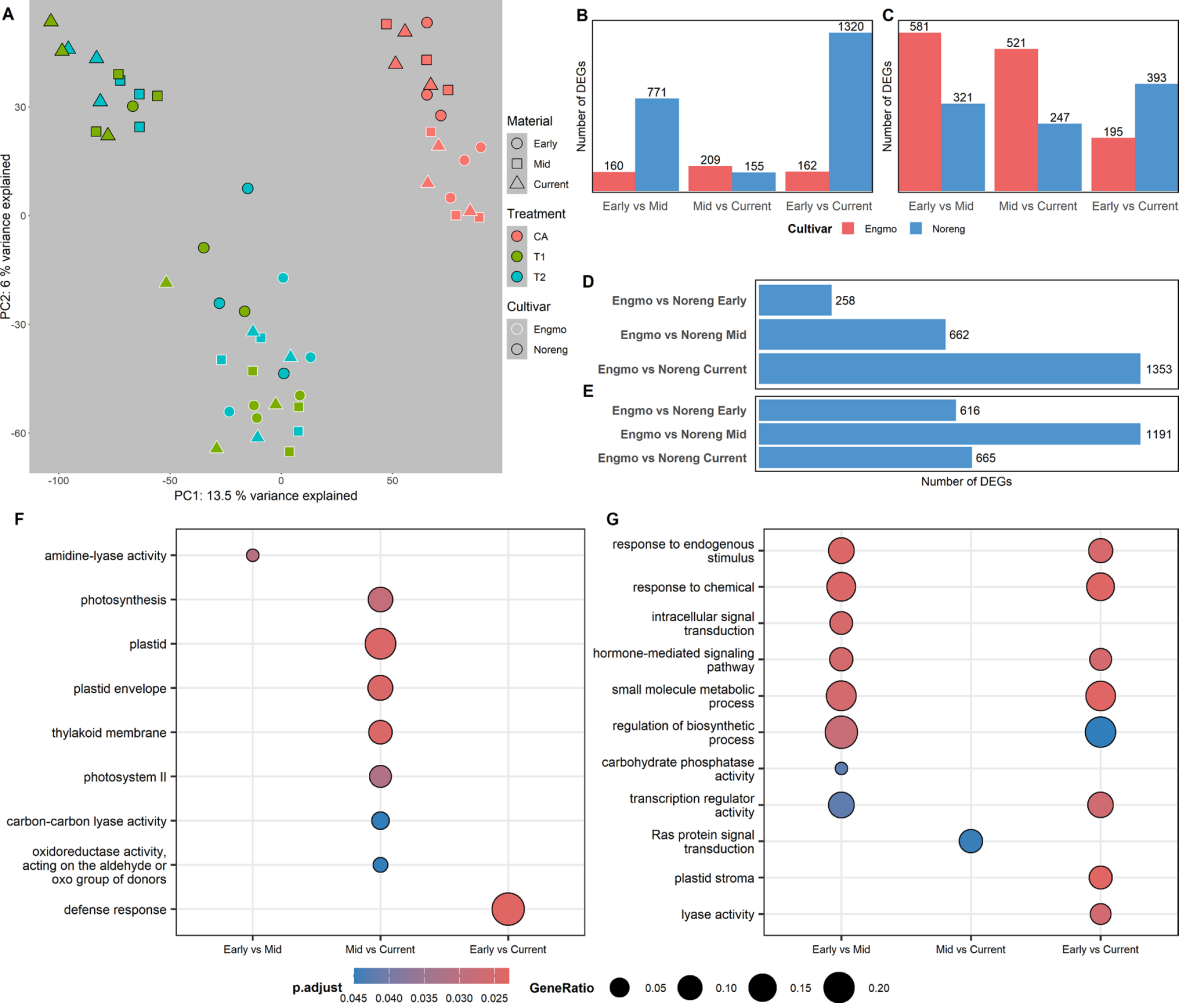
**A** PCA plot based on gene expression under freezing stress. CA = cold acclimation, *T*1 = − 18 °C (below LT_50_), *T*2 = − 24 °C (above LT_50_). Early = 1988–98, mid = 2003–10, current = 2020. **B** Differentially expressed genes (DEGs) between temporal populations of respective cultivars under freezing stress and **C** ice encasement stress. **D** DEGs between cultivars at a given temporal population under freezing stress and **E** ice encasement stress. **F** Highly enriched GO terms between populations of Engmo and **G** Noreng. The gene set for GO enrichment analysis is the union of DEGs identified across all comparisons between populations. For example, the gene set of Early vs Mid is a union of DEGs from Early CA vs Mid CA, Early T1 vs Mid T1, and Early T2 vs Mid T2 of a given cultivar

PCA based on 70,526 genes retained after filtering for low expression separated CA and treatment samples (*T*1 and *T*2) of both cultivars along PC1 (13.5% variation) (Fig. 1A). PC2 (6% variation) appears to separate samples of Engmo and Noreng. Interestingly, the *T*1 and *T*2 samples of Early Noreng population appear to cluster closer to the Engmo populations than to the rest of the Noreng populations (Fig. 1A). Contrasts between temporal populations in both cultivars identified several genes with differential expression under freezing stress. The number of DEGs between populations of Noreng was higher than the number of DEGs between populations of Engmo (Fig. 1B, 1D). In particular, the number of DEGs between populations of Noreng appears to increase with the temporal (time) difference between the populations, i.e., Early vs Mid < Early vs Current > Mid vs Current (Fig. 1B). Furthermore, there was a gradual increase in the number of DEGs between the cultivars over time, i.e., Current > Mid > Early (Fig. 1D), suggesting divergence in freezing stress gene expression between the cultivars and many of the DEGs between populations of Noreng overlap with the DEGs between the populations of Engmo and Noreng.

GO analysis on sets of DEGs between populations of Engmo revealed that the majority of enriched terms were in DEGs between Mid vs Current (Fig. 1F). Further inspection of the expression of DEGs between Engmo did not reveal any interesting patterns. Among the GO terms identified as enriched between populations of Noreng, many were common between Early vs Mid and Early vs Current (Fig. 1G). Moreover, genes coding for dehydration-responsive element binding 1 (DREB) transcription factors (TFs), fructan 6-exohydrolase, beta-fructofuranosidase (invertase), flowering locus T-like and zinc finger LSD1 appear to have lower expression under freezing stress in Mid and Current compared to Early population of Noreng, while genes encoding cold-responsive protein kinase (CRPK) 1, ethylene-responsive transcription factors (ERF), DAHP synthetase, and NAC 6 TFs were among genes with higher expression in Mid and Current compared to Early population in Noreng. Boxplots of genes displaying shifts over time are attached as supplementary figures (Figure S2–S5).

### Differential gene expression under ice encasement

The PC1 explaining 40% variation separated CA samples and ice encased samples (*T*1 and *T*2), and PC2 explaining 9.6% variation separated *T*1 and *T*2 samples (Figure S6). The first four PC axes did not cluster the samples based on cultivars or populations. Similar to under freezing stress, the number of DEGs appears to increase with the temporal differences between the populations of Noreng (Early vs Mid < Early vs Current > Mid vs Current) (Fig. 1C). In the case of Engmo, the number of DEGs in Early vs Mid and Mid vs Current populations were higher than in Early vs Current (Fig. 1C). In addition, the number of DEGs between Engmo and Noreng was higher in Mid population compared to Early or Current populations (Fig. 1E). This suggests both of the previous observations could be due to the origin (adaptation background) of Engmo Mid population. A comprehensive inspection of DEGs lists did not identify any genes with expression patterns resembling shifts. Furthermore, there were no enriched GO terms among the DEGs between populations of either Engmo or Noreng. Given this, we decided to emphasize the gene expression changes over time under freezing stress.

### Allele frequency changes and genetic diversity estimates

**Fig. 2:**
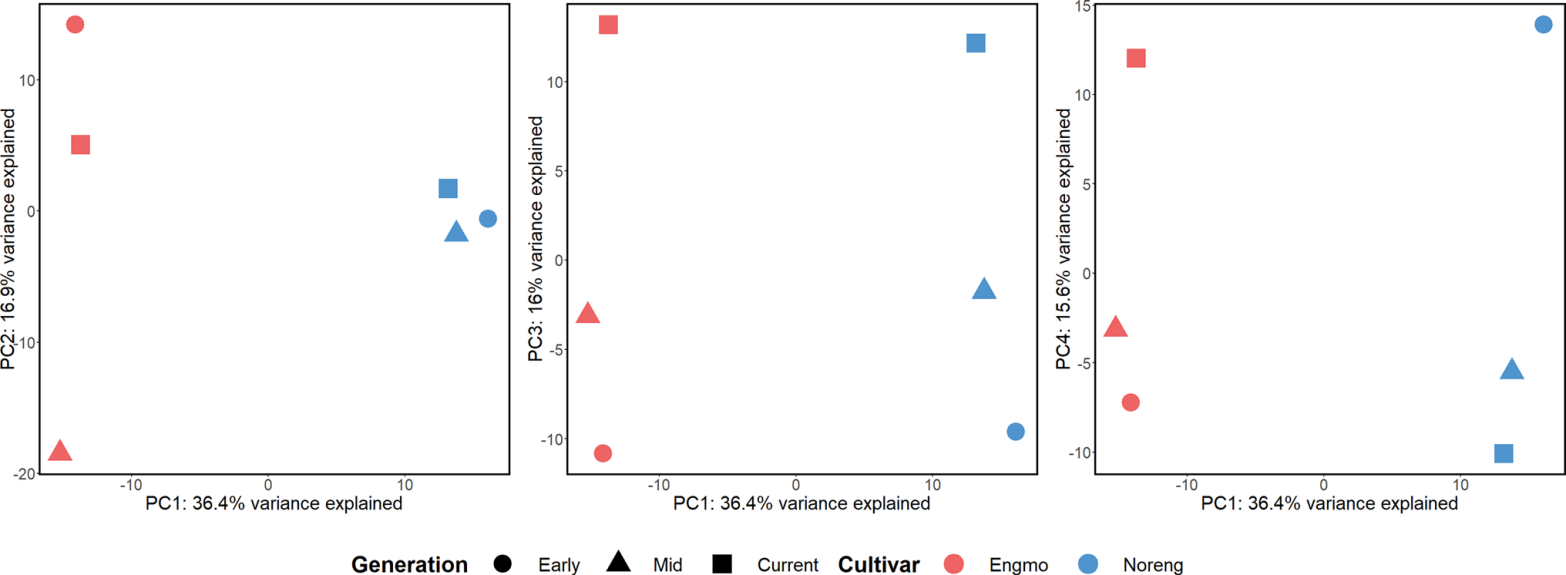
PCA plot based on 93,338 common SNPs across all populations. PC1 is the x-axis in all plots, and PC2, PC3, and PC4 are the y-axis in the left, middle & right plots, respectively

The alignment rate of SLAF-seq reads was > 97% for all samples. PCA was performed based on allele frequencies at 93,338 SNPs (loci) common across all populations after filtering for low read depth, read support of minor allele, and minor allele frequency. The first four principal components revealed interesting insights. PC1 (36.4% variance) separated the cultivars, PC2 (16.9% variance) separated the Engmo Mid from the rest of the populations of both cultivars, PC3 (16% variance) ordered the Early, Mid and Current populations of both cultivars and lastly, PC4 (15.6% variance) ordered the Early, Mid and Current populations of the cultivars in opposite direction (Fig. 2). 2976 among the 5148 SNPs (top 5%) selected based on *|ΔAF|*, were located within 10 kilobases (kb) flanking regions of protein-coding genes. Moreover, 81 genes identified as DE between populations or cultivars under freezing stress, which include DREB 1B, ERF ABA repressor 1 (ABR1), bHLH 148-like, and galactinol synthase 2, were within the 10 kb flanking region of those SNPs selected based on *|ΔAF|*. After filtering (at least five valid SNPs), a total of 27,808 windows common across all populations in both cultivars were used to calculate the genome-wide nucleotide diversity (*θπ*), watterson’s θ (*θw*), and tajimas D (Table 2).

**Table 2:** Genome-wide genetic diversity estimates in temporal populations of Engmo and Noreng. Values are the mean ± standard error (SE) of per-window estimates across the filtered genomic windows. Pairwise Welch’s *t*-tests were performed within each cultivar, with Benjamini–Hochberg FDR correction across all 18 pairwise comparisons. Populations sharing the same superscript do not differ significantly at FDR-adjusted *p* < 0.05

		Early	Mid	Current
Nucleotide diversity (*θπ*)	Engmo	0.00955^ab^ ± 4.81e−5	0.00965^a^ ± 4.83e−5	0.00942^b^ ± 4.75e−5
	Noreng	0.00984^a^ ± 4.87e−5	0.00980^a^ ± 4.86e−5	0.00973^a^ ± 4.86e−5
Watterson’s *θ* (*θw*)	Engmo	0.00884^ab^ ± 3.85e−5	0.00872^a^ ± 3.79e−5	0.00886^b^ ± 3.87e − 5
	Noreng	0.00877^a^ ± 3.82e−5	0.00879^a^ ± 3.84e−5	0.00883^a^ ± 3.85e−5
Tajima’s *D*	Engmo	0.23^a^ ± 7.04e−3	0.31^b^ ± 7.15e−3	0.18^c^ ± 7.02e−3
	Noreng	0.37^a^ ± 7.11e−3	0.34^b^ ± 7.04e−3	0.30^c^ ± 6.99e−3

Nucleotide diversity (*θπ*) was consistently higher in the synthetic cultivar Noreng than in the landrace Engmo across all three temporal populations (Table 2) (Table 2). The genome-wide F_st_ between populations of Engmo were 0.031 (Early vs Mid), 0.029 (Mid vs Current), and 0.026 (Early vs Current), while they were 0.027 (Early vs Mid), 0.026 (Mid vs Current), and 0.028 (Early vs Current) between populations of Noreng. *F*_st_ analysis identified 303 and 268 windows with *F*_st_ ≥ 0.15 (*p*.adj < 0.05) between populations of Engmo and Noreng, respectively. Furthermore, 14 of 529 genes overlapping with *F*_st_ windows in Noreng were differentially expressed between populations of Noreng under freezing stress, while only two of the 575 genes overlapping with *F*_st_ windows of Engmo were differentially expressed between populations of Engmo. Among the 14 DEGs overlapping with the *F*_st_ windows in Noreng, three genes (Ppra_g106057, Ppra_g106058, Ppra_g106059) coding for cortical cell-delineating protein were observed to be downregulated > twofold in Mid and Current compared to early population in Noreng. GO enrichment analysis of the genes overlapping with the F_st_ windows in either of the cultivars did not yield any enriched terms.

## Discussion

### Differences in gene expression under freezing stress

Based on the clustering of the samples along the first two principal component axes (Fig. 1A) coupled with an increase in the number of DEGs between populations of Noreng over time (Fig. 1B), it is evident that Noreng is experiencing shifts in freezing stress gene expression. We define gene expression shift as a non-random change in gene expression between temporal populations represented by expression patterns such as Early > Mid ≥ Current or Early < Mid ≤ Current. Furthermore, the huge overlap of DEGs between populations of Noreng with the DEGs between populations of Engmo and Noreng suggests that divergence in freezing stress gene expression between the cultivars is primarily due to shifts in Noreng. The cultivars appear to have a contrasting relationship in terms of gene expression differences and freezing stress survival rates over time (Fig. 1A, B, Table 1). Despite huge differences in freezing stress gene expression, there were no significant differences in survival rates among Noreng populations. Contrastingly, there was a significant decline in survival (Mid and Current compared to Early) in Engmo populations, which had relatively few differences in freezing stress gene expression. This observation raises an important question: Do the observed gene expression shifts in Noreng facilitate its adaptation to novel stressful conditions under changing climate? The answer to this question might lie in the functional role of genes identified as experiencing shifts in abiotic stress responses. We were particularly interested in genes that display non-random or directional changes in gene expression over time, as they may be indicative of adaptive responses to changing climatic conditions.

In plants, temperature and light stimuli are the key regulators of the circadian clock (Hirschie Johnson et al. 2003; Harmer 2009; Avello et al. 2019; MacKinnon et al. 2020), which is known to play a crucial role in several biological processes and biotic and abiotic stress responses (Fowler et al. 2005; Covington et al. 2008; Dong et al. 2011; Seo and Mas 2015). Though photoperiod will remain constant, the increase in autumn and winter temperatures at higher northern latitudes (Uleberg et al. 2014; Bjerke et al. 2015; Dyrrdal et al. 2020) is expected to subject the plants to different temperature x photoperiod regimes during cold acclimation in autumn and freezing conditions in winter, which could negatively affect their winter survival (Gray et al. 1997; Rapacz et al. 2014; Dalmannsdottir et al. 2017; Liu et al. 2019; Ahres et al. 2020). Moreover, the induction of CBF1/DREB1 is known to be gated by Circadian Clock-Associated 1 (CCA1) and Late Elongated Hypocotyl (LHY), the core circadian clock components (Fowler et al. 2005; Dong et al. 2011). In the current study, the expression of genes linked to the circadian rhythm, flowering and light perception such as LHY (Li et al. 2022), Flowering Locus T (Pin and Nilsson 2012), ultraviolet-B receptor 8 (UVR8) (Fehér et al. 2011), Light-regulated protein 1 (LIR1) (Ciannamea et al. 2007; Yang et al. 2016) had significantly lower expression in Noreng Mid and Current populations, while the expression of MADS-box 51 (MADS51) (Kim et al. 2007) was higher in Mid and Current populations under freezing stress (Figure S2). Based on the gene expression shifts and freezing tolerance, we suspect genes linked to might play a role in fine-tuning cold acclimation and freezing stress responses under novel light x temperature regimes.

Transcription factors tune the physiological processes in response to changes in the surrounding environment and thus play a crucial role in adaptation to stressful conditions. Notably, genes which are known to (negatively) regulate ethylene biosynthesis, abscisic acid (ABA) responses and ethylene responses i.e., ERF 109 TF, ethylene receptor 2 (ETR2), reversion to ethylene sensitivity 1 (RTE1), EIN3-binding F-box protein 1-like (EBF-1 like), and abscisic acid repressor 1 (ABR1) (Gagne et al. 2004; Pandey et al. 2005; Zhou et al. 2007; Wuriyanghan et al. 2009; Yu et al. 2017) have higher expression in Mid and Current compared to Early population of Noreng under freezing conditions (Figure S3). Functional analysis identified the GO term *hormone-mediated signaling pathway* (GO:0009755) and *transcription regulator activity* (GO:0140110) as highly enriched among DEGs between Early vs Mid and Early vs Current populations of Noreng, indicating the shifts in expression of transcription factors associated with this term are not random. This observation suggests that phytohormone (ethylene and ABA) mediated transcriptional regulation might facilitate adaptation to highly variable winter weather conditions.

Moreover, the expression of cold-responsive protein kinase 1 (CRPK1), a negative regulator of freezing tolerance by modulating the expression of DREB1 post-translationally (Liu et al. 2017), was higher in Mid and Current populations compared to the Early population in Noreng. In line with the previous observation, genes coding for DREB 1B, DREB 1H TFs have significantly lower expression at *T*1 and *T*2 in Mid and Current compared to Early populations of Noreng, while not significant, a similar pattern is observed between Current and Early populations of Engmo (Figure S4). The decline in freezing tolerance in populations of both cultivars could be partly explained by the lower expression of DREB1, whose overexpression is known to confer higher tolerance to drought and freezing stress (Jaglo-Ottosen et al. 1998; Ito et al. 2006). However, the expression pattern of DREB1 does not explain the significant decline in survival (at − 21 °C) in the Engmo populations and relatively stable survival rates in Noreng populations.

Programmed cell death (PCD) is one of the adaptation strategies that plants use to mitigate the effects of stressors on their fitness and survival (Locato and De Gara 2018; Burke et al. 2020). Studies from Hong et al. (2017), and Yang et al. (2024) reported that PCD under cold/freezing stress led to enhanced recovery and survival. In the current study, the expression of zinc finger LSD1, a negative regulator of cell death (Dietrich et al. 1997) was lower in the Mid and Current compared to Early population of Noreng, while the expression of NAC6 transcription factor, a positive regulator of cell death (Faria et al. 2011) was higher in the Mid and Current compared to the Early population of Noreng at *T*1 and *T*2 (Figure S5). This observation indicates that programmed cell death (PCD) might be an important strategy for survival by limiting the extent of winter injuries (Hong et al. 2017; Burke et al. 2020) under changing climate. Though the reduced persistence may require frequent resowing, leading to an increased cost of cultivation in the short run, it may be beneficial in the long run as it could aid in faster adaptation by reducing generation time and limiting generation overlap (Kuparinen et al. 2010; Anderson et al. 2011; Yamamichi et al. 2019; Moran 2020).

Interestingly, the temporal populations of cultivar Engmo appear to have very little gene expression differences among them under freezing stress, despite a significant decline in freezing tolerance over time (Table 1), and the Canadian origin of the Engmo Mid population. The few DEGs identified between populations of Engmo did not exhibit patterns indicating shifts in gene expression, suggesting they might be random. After Engmo was reintroduced into the market in 2017, its certified seed production was based in the south-east of Norway. The lack of shifts in gene expression over Engmo populations despite the differences in SNP allelic frequencies (Fig. 2) and the reduced freezing tolerance in the Mid and Current populations (Table 1) are hard to explain. The reduction in freezing tolerance could be an effect of the seed multiplication environment, with the Early generation being multiplied in the north (~ 69°N) while the Mid and Current populations were multiplied at lower latitudes (~ 46°N and 59°N, respectively) under quite different photoperiods, temperatures, and winter climates. The maintenance of genetic polymorphism and the stable gene expression of selected candidate genes could be a result of balancing selection due to reproduction in spatially variable environments (Hedrick 2006). Another explanation might be that the large genetic variation and population buffering capacity in populations like these counteract any effects of selection.

### Differences in gene expression under ice encasement

Among the genes identified as differentially expressed between populations within cultivars under ice encasement, none displayed expression patterns resembling shifts, indicating that most of the differential expression could be stochastic changes. Based on the number of DEGs between Engmo and Noreng in Early and Current populations (Fig. 1E), it appears that either the cultivars did not diverge in terms of gene expression under ice encasement or both experienced changes in the same direction. However, very few DEGs identified by contrasts between populations of a cultivar (Engmo Early vs Engmo Current or Noreng Early vs Noreng Current) overlap with the DEGs between Early or Current populations (Engmo Early vs Noreng Early and Engmo Current vs Noreng Current) of the cultivars. Moreover, these overlapping genes have very low expression and do not show any interesting expression pattern between temporal populations of a cultivar or between cultivars. This suggests that both cultivars experienced little to no changes in ice encasement gene expression over the years, and the DEGs identified could be due to random changes in gene expression. One explanation for the lack of shifts in ice encasement stress over time could be weak selection pressure. Although the frequency of ice encasement has risen in recent years (Bjerke et al. 2015; Dyrrdal et al. 2020), they remain less prevalent than freezing stress conditions, which occur consistently every winter. The ice encasement stress under lab conditions is different from the natural field conditions and much more severe (Gudleifsson 2010). Plants are selected for ice encasement under natural field conditions; thus, we suspect the differences in experimental conditions in the lab compared to the field may have hindered the detection of adaptive stress responses, if there were any.

### Allele frequency changes in temporal populations

**Fig. 3:**
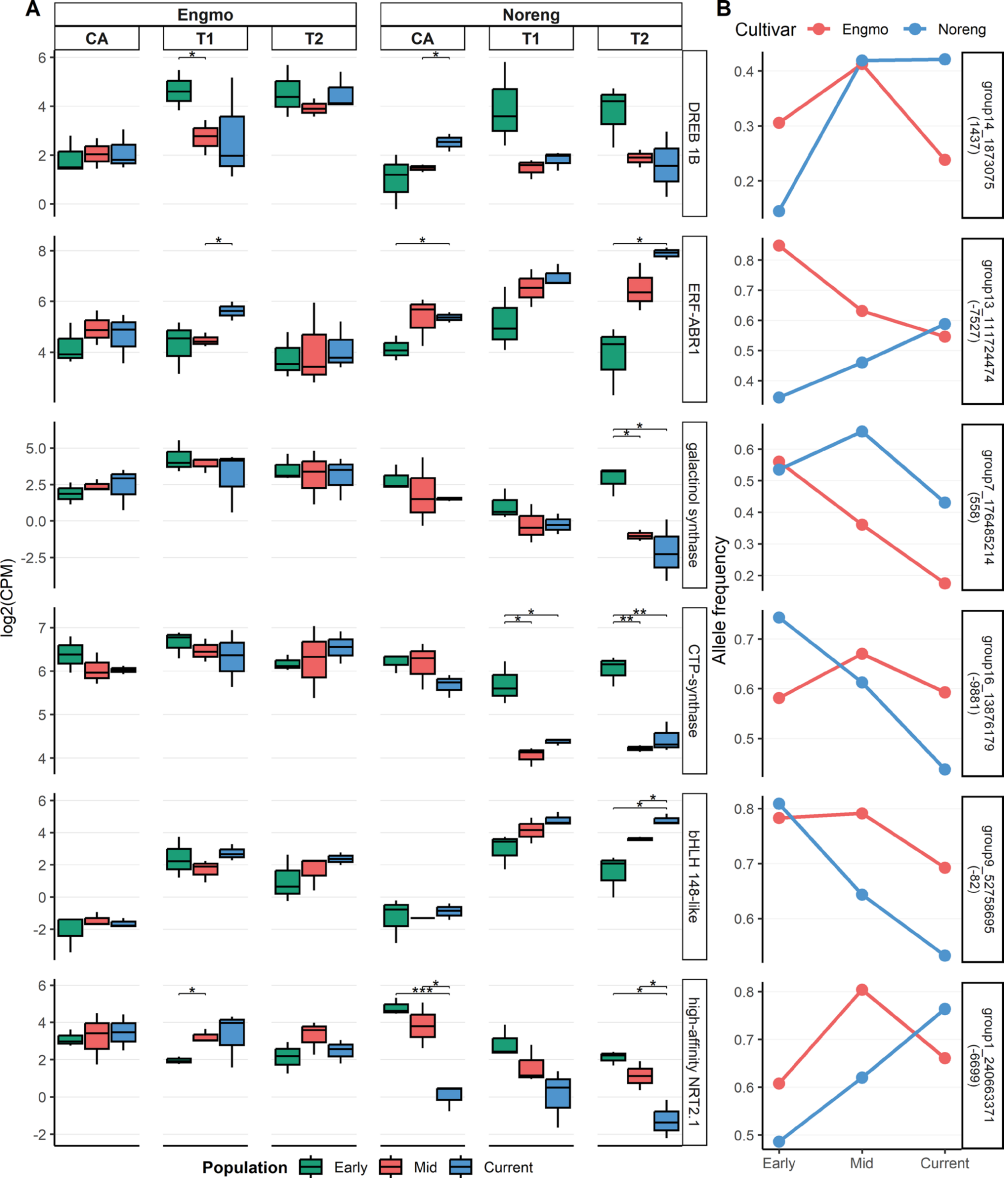
**A** Genes identified as differentially expressed under freezing stress that are in the flanking region (10 kb) of SNPs among the 95th percentile of |ΔAF|. CA = cold acclimation, T1 = − 18 °C, T2 = − 24 °C. Early = 1988–98, mid = 2003–10, current = 2020. The genes are identified as differentially expressed between populations of a cultivar or between cultivars. The statistical significance in plot A is based on a *t*-test between populations at a given treatment. **B** Allele frequencies of the SNPs in the flanking region of genes. Strip text in plot B denotes SNP ID and distance to the gene. + distance = upstream of the gene, and − distance = downstream of the gene

The allele frequency estimates separated the populations according to their genetic backgrounds along the first three principal component axes (Fig. 2, Figure S7). This suggests that SLAF-seq based on DNA pooled from multiple individuals of the populations is a reliable and cost-effective method for studying population structure, diversity, and allele frequency changes in species with large genomes. The ordination of Early, Mid, and Current populations along PC3 and PC4 (Fig. 2) indicates that timothy cultivars in the current study are experiencing directional as well as diverging allele frequency changes. In general, there was a decline in genome-wide *θπ*, *θw,* and tajimas D over time in both cultivars (Table 2), likely due to reduced allelic variation driven by purifying selection. Based on the known history of the cultivars, the landrace Engmo was expected to be more genetically diverse compared to the synthetic cultivar Noreng (based on 14 genotypes), but our genetic diversity estimates indicate otherwise (Table 2). There are very few studies comparing genetic variation in comparable types of forage grass cultivars. However, a comparison of genetic variation in perennial ryegrass (Lolium perenne L.) ecotypes and cultivars using 2199 SNP markers found similar results. Within population variation was larger in commercially bred (synthetic) forage cultivars (72 to 82%) compared with ecotypes (70%) (Blackmore et al. 2016). The landrace Engmo in this context is comparable to ecotypes. Another explanation of the larger genetic variation in Noreng could be that it is a synthetic made from 10 Engmo and 4 Grindstad genotypes. The diverse adaptation and origin of Engmo (northern adapted and northern origin) and Grindstad (southern adapted, Scottish origin) could explain the larger genetic variation in Noreng. Interestingly, SNPs with the biggest *|ΔAF|* were observed in proximity (10 kb) to genes like DREB 1B, ABR1, and bHLH 148-like (Fig. 3). We suspect that these genetic changes may have contributed to the differential expression of these genes between populations of Noreng. The readers should note that our speculation is based on the observed correlation and does not imply a causal relationship between allele frequency and gene expression shifts.

## Limitations

In the current study, two biological replicates (two plants/replicate) were used to investigate gene expression differences, while at least three replicates are recommended for transcriptomic studies. To ensure robustness, a pseudo-replication and log-fold change threshold were used. In addition, the expression of the genes in the discussion section was replicated at both freezing stress treatments (*T*1 and *T*2), ensuring the shifts detected are statistically robust. In resurrection studies, plants from the refresher generation are commonly used to minimize the storage and maternal effects (Franks et al. 2018). In the current study, plants raised directly from seedlots were used to study gene expression differences between the populations. Although maternal or storage effects may have contributed to the observed gene expression patterns, it is highly unlikely that these effects alone were responsible for the observed directional shifts in the expression of genes discussed in the results and discussion.

## Conclusion

The cultivars in the study appear to diverge in terms of freezing stress gene expression, driven mainly by gene expression shifts in Noreng. Several genes displaying patterns indicating shifts between populations of Noreng are known to play an important role in signaling and stress responses. Weak selection pressure could be the reason behind the lack of gene expression shifts under ice encasement. The cultivars are experiencing directional as well as diverging allele frequency changes. The resurrection approach combined with comparative transcriptomic analysis is a powerful approach to studying how plants tune their gene expression to adapt to changing climatic conditions. The results also demonstrate that pool-seq based SLAF-seq is a reliable and cost-effective approach to study genetic diversity in species with large genomes with high intrapopulation variation.

## Supplementary Information

Below is the link to the electronic supplementary material.

Supplementary file1 (DOCX 1197 KB)

## Data Availability

The RNA-seq & SLAF data generated are available at Array Express EBI with reference numbers E-MTAB-14732 and E-MTAB-16539, respectively.

## References

[CR1] Ahres M, Gierczik K, Boldizsár Á, Vítámvás P, Galiba G (2020) Temperature and light-quality-dependent regulation of freezing tolerance in barley. Plants 9:83. 10.3390/plants9010083 31936533 10.3390/plants9010083 PMC7020399

[CR2] Aitken SN, Yeaman S, Holliday JA, Wang T, Curtis-McLane S (2008) Adaptation, migration or extirpation: climate change outcomes for tree populations. Evol Appl 1:95–111. 10.1111/j.1752-4571.2007.00013.x 25567494 10.1111/j.1752-4571.2007.00013.x PMC3352395

[CR3] Anderson JT, Willis JH, Mitchell-Olds T (2011) Evolutionary genetics of plant adaptation. Trends Genet 27:258–266. 10.1016/j.tig.2011.04.001 21550682 10.1016/j.tig.2011.04.001 PMC3123387

[CR4] Anderson JT, Inouye DW, McKinney AM, Colautti RI, Mitchell-Olds T (2012a) Phenotypic plasticity and adaptive evolution contribute to advancing flowering phenology in response to climate change. Proc R Soc B Biol Sci 279:3843–3852. 10.1098/rspb.2012.1051 10.1098/rspb.2012.1051 PMC3415914 22787021

[CR5] Anderson JT, Panetta AM, Mitchell-Olds T (2012b) Evolutionary and ecological responses to anthropogenic climate change: update on anthropogenic climate change. Plant Physiol 160:1728–1740. 10.1104/pp.112.206219 23043078 10.1104/pp.112.206219 PMC3510106

[CR6] Andrews S (2010) FastQC a quality control tool for high throughput sequence data : Babraham bioinformatics. Available at: https://www.bioinformatics.babraham.ac.uk/projects/fastqc/. Accessed 27 Jun 2022

[CR7] Avello PA, Davis SJ, Ronald J, Pitchford JW (2019) Heat the clock: entrainment and compensation in *Arabidopsis* circadian rhythms. J Circadian Rhythms 17:5. 10.5334/jcr.179 31139231 10.5334/jcr.179 PMC6524549

[CR8] Becklin KM, Anderson JT, Gerhart LM, Wadgymar SM, Wessinger CA, Ward JK (2016) Examining plant physiological responses to climate change through an evolutionary lens. Plant Physiol. 10.1104/pp.16.00793 27591186 10.1104/pp.16.00793 PMC5047093

[CR9] Bjerke JW, Tømmervik H, Zielke M, Jørgensen M (2015) Impacts of snow season on ground-ice accumulation, soil frost and primary productivity in a grassland of sub-Arctic Norway. Environ Res Lett 10:095007. 10.1088/1748-9326/10/9/095007

[CR10] Blackmore T, Thorogood D, Skøt L, McMahon R, Powell W, Hegarty M (2016) Germplasm dynamics: the role of ecotypic diversity in shaping the patterns of genetic variation in *Lolium perenne*. Sci Rep 6:22603. 10.1038/srep22603 26935901 10.1038/srep22603 PMC4776279

[CR11] Buchfink B, Reuter K, Drost H-G (2021) Sensitive protein alignments at tree-of-life scale using DIAMOND. Nat Methods 18:366–368. 10.1038/s41592-021-01101-x 33828273 10.1038/s41592-021-01101-x PMC8026399

[CR12] Burke R, Schwarze J, Sherwood OL, Jnaid Y, McCabe PF, Kacprzyk J (2020) Stressed to death: the role of transcription factors in plant programmed cell death induced by abiotic and biotic stimuli. Front Plant Sci 11:1235. 10.3389/fpls.2020.01235 32903426 10.3389/fpls.2020.01235 PMC7434935

[CR13] Chen I-C, Hill JK, Ohlemüller R, Roy DB, Thomas CD (2011) Rapid range shifts of species associated with high levels of climate warming. Science 333:1024–1026. 10.1126/science.1206432 21852500 10.1126/science.1206432

[CR14] Chen S, Zhou Y, Chen Y, Gu J (2018) fastp: an ultra-fast all-in-one FASTQ preprocessor. Bioinformatics 34:i884–i890. 10.1093/bioinformatics/bty560 30423086 10.1093/bioinformatics/bty560 PMC6129281

[CR15] Chevin L-M, Lande R, Mace GM (2010) Adaptation, plasticity, and extinction in a changing environment: towards a predictive theory. PLoS Biol 8:e1000357. 10.1371/journal.pbio.1000357 20463950 10.1371/journal.pbio.1000357 PMC2864732

[CR16] Ciannamea S, Jensen CS, Agerskov H, Petersen K, Lenk I, Didion T et al (2007) A new member of the *LIR* gene family from perennial ryegrass is cold-responsive, and promotes vegetative growth in *Arabidopsis*. Plant Sci 172:221–227. 10.1016/j.plantsci.2006.08.011

[CR17] Covington MF, Maloof JN, Straume M, Kay SA, Harmer SL (2008) Global transcriptome analysis reveals circadian regulation of key pathways in plant growth and development. Genome Biol 9:R130. 10.1186/gb-2008-9-8-r130 18710561 10.1186/gb-2008-9-8-r130 PMC2575520

[CR18] Cunze S, Heydel F, Tackenberg O (2013) Are plant species able to keep pace with the rapidly changing climate? PLoS ONE 8:e67909. 10.1371/journal.pone.0067909 23894290 10.1371/journal.pone.0067909 PMC3722234

[CR19] Czech L, Spence JP, Expósito-Alonso M (2024) Grenedalf: population genetic statistics for the next generation of pool sequencing. Bioinformatics 40:btae508. 10.1093/bioinformatics/btae508 39185959 10.1093/bioinformatics/btae508 PMC11357794

[CR20] Dalmannsdottir S, Jørgensen M, Rapacz M, Østrem L, Larsen A, Rødven R et al (2017) Cold acclimation in warmer extended autumns impairs freezing tolerance of perennial ryegrass (*Lolium perenne*) and timothy (*Phleum pratense*). Physiol Plantarum 160:266–281. 10.1111/ppl.12548 10.1111/ppl.12548 28144950

[CR21] Danecek P, Bonfield JK, Liddle J, Marshall J, Ohan V, Pollard MO et al (2021) Twelve years of SAMtools and BCFtools. GigaScience 10:giab008. 10.1093/gigascience/giab008 33590861 10.1093/gigascience/giab008 PMC7931819

[CR22] Davis MB, Shaw RG (2001) Range shifts and adaptive responses to quaternary climate change. Science 292:673–679. 10.1126/science.292.5517.673 11326089 10.1126/science.292.5517.673

[CR23] DeWitt TJ, Sih A, Wilson DS (1998) Costs and limits of phenotypic plasticity. Trends Ecol Evol 13:77–81. 10.1016/S0169-5347(97)01274-3 21238209 10.1016/s0169-5347(97)01274-3

[CR24] Dietrich RA, Richberg MH, Schmidt R, Dean C, Dangl JL (1997) A novel zinc finger protein is encoded by the arabidopsis *LSD1* gene and functions as a negative regulator of plant cell death. Cell 88:685–694. 10.1016/S0092-8674(00)81911-X 9054508 10.1016/s0092-8674(00)81911-x

[CR25] Dobin A, Davis CA, Schlesinger F, Drenkow J, Zaleski C, Jha S et al (2013) STAR: ultrafast universal RNA-seq aligner. Bioinformatics 29:15–21. 10.1093/bioinformatics/bts635 23104886 10.1093/bioinformatics/bts635 PMC3530905

[CR26] Dong MA, Farré EM, Thomashow MF (2011) Circadian clock-associated 1 and late elongated hypocotyl regulate expression of the C-repeat binding factor (CBF) pathway in *Arabidopsis*. Proc Natl Acad Sci U S A 108:7241–7246. 10.1073/pnas.1103741108 21471455 10.1073/pnas.1103741108 PMC3084081

[CR27] Dyrrdal AV, Isaksen K, Jacobsen JKS, Nilsen IB (2020) Present and future changes in winter climate indices relevant for access disruptions in Troms, Northern Norway. Nat Hazards Earth Syst Sci 20:1847–1865. 10.5194/nhess-20-1847-2020

[CR28] Faria JA, Reis PA, Reis MT, Rosado GL, Pinheiro GL, Mendes GC et al (2011) The NAC domain-containing protein, GmNAC6, is a downstream component of the ER stress- and osmotic stress-induced NRP-mediated cell-death signaling pathway. BMC Plant Biol 11:129. 10.1186/1471-2229-11-129 21943253 10.1186/1471-2229-11-129 PMC3193034

[CR29] Fehér B, Kozma-Bognár L, Kevei É, Hajdu A, Binkert M, Davis SJ et al (2011) Functional interaction of the circadian clock and UV RESISTANCE LOCUS 8-controlled UV-B signaling pathways in *Arabidopsis thaliana*. Plant J 67:37–48. 10.1111/j.1365-313X.2011.04573.x 21395889 10.1111/j.1365-313X.2011.04573.x

[CR30] Fowler SG, Cook D, Thomashow MF (2005) Low temperature induction of *Arabidopsis* CBF1, 2, and 3 is gated by the circadian clock. Plant Physiol 137:961–968. 10.1104/pp.104.058354 15728337 10.1104/pp.104.058354 PMC1065397

[CR31] Fox J, Weisberg S, Price B (2001) Car: companion to applied regression. 3.1–2. 10.32614/CRAN.package.car

[CR32] Franks SJ (2011) Plasticity and evolution in drought avoidance and escape in the annual plant *Brassica rapa*. New Phytol 190:249–257. 10.1111/j.1469-8137.2010.03603.x 21210818 10.1111/j.1469-8137.2010.03603.x

[CR33] Franks SJ, Avise JC, Bradshaw WE, Conner JK, Etterson JR, Mazer SJ et al (2008) The Resurrection Initiative: storing ancestral genotypes to capture evolution in action. Bioscience 58:870–873. 10.1641/B580913

[CR34] Franks SJ, Weber JJ, Aitken SN (2014) Evolutionary and plastic responses to climate change in terrestrial plant populations. Evol Appl 7:123–139. 10.1111/eva.12112 24454552 10.1111/eva.12112 PMC3894902

[CR35] Franks SJ, Kane NC, O’Hara NB, Tittes S, Rest JS (2016) Rapid genome-wide evolution in *Brassica rapa* populations following drought revealed by sequencing of ancestral and descendant gene pools. Mol Ecol 25:3622–3631. 10.1111/mec.13615 27072809 10.1111/mec.13615 PMC4963267

[CR36] Franks SJ, Hamann E, Weis AE (2018) Using the resurrection approach to understand contemporary evolution in changing environments. Evol Appl 11:17–28. 10.1111/eva.12528 29302269 10.1111/eva.12528 PMC5748528

[CR37] Gagne JM, Smalle J, Gingerich DJ, Walker JM, Yoo S-D, Yanagisawa S et al (2004) Arabidopsis EIN3-binding F-box 1 and 2 form ubiquitin-protein ligases that repress ethylene action and promote growth by directing EIN3 degradation. Proc Natl Acad Sci U S A 101:6803–6808. 10.1073/pnas.0401698101 15090654 10.1073/pnas.0401698101 PMC404126

[CR38] Gray GR, Chauvin LP, Sarhan F, Huner N (1997) Cold acclimation and freezing tolerance (a complex interaction of light and temperature). Plant Physiol 114:467–474. 10.1104/pp.114.2.467 12223720 10.1104/pp.114.2.467 PMC158326

[CR39] Gudleifsson B (2010) Ice tolerance and metabolite accumulation of herbage crops in Iceland and impact of climate change. Icel Agric Sci 23:111–122

[CR40] Harmer SL (2009) The circadian system in higher plants. Annu Rev Plant Biol 60:357–377. 10.1146/annurev.arplant.043008.092054 19575587 10.1146/annurev.arplant.043008.092054

[CR41] Hart EH, Christofides SR, Davies TE, Rees Stevens P, Creevey CJ, Müller CT et al (2022) Forage grass growth under future climate change scenarios affects fermentation and ruminant efficiency. Sci Rep 12:4454. 10.1038/s41598-022-08309-7 35292703 10.1038/s41598-022-08309-7 PMC8924208

[CR42] Havstad LT, Aamlid TS (2013) Influence of harvest time and storage location on the longevity of timothy (*Phleum pratense* L.) seed. Acta Agric Scand B Soil Plant Sci 63:453–459. 10.1080/09064710.2013.798426

[CR43] Hedrick PW (2006) Genetic polymorphism in heterogeneous environments: the age of genomics. Annu Rev Ecol Evol Syst 37:67–93. 10.1146/annurev.ecolsys.37.091305.110132

[CR44] Hirschie Johnson C, Elliott JA, Foster R (2003) Entrainment of circadian programs. Chronobiol Int 20:741–774. 10.1081/CBI-120024211 14535352 10.1081/cbi-120024211

[CR45] Hoffmann AA, Sgrò CM (2011) Climate change and evolutionary adaptation. Nature 470:479–485. 10.1038/nature09670 21350480 10.1038/nature09670

[CR46] Hong JH, Savina M, Du J, Devendran A, Ramakanth KK, Tian X et al (2017) A sacrifice-for-survival mechanism protects root stem cell niche from chilling stress. Cell 170:102-113.e14. 10.1016/j.cell.2017.06.002 28648662 10.1016/j.cell.2017.06.002

[CR47] IPCC (2014) Climate change 2013–the physical science basis: working group I contribution to the fifth assessment report of the intergovernmental panel on climate change, 1st edn. Cambridge University Press

[CR48] Ito Y, Katsura K, Maruyama K, Taji T, Kobayashi M, Seki M et al (2006) Functional analysis of rice DREB1/CBF-type transcription factors involved in cold-responsive gene expression in transgenic rice. Plant Cell Physiol 47:141–153. 10.1093/pcp/pci230 16284406 10.1093/pcp/pci230

[CR49] Jaglo-Ottosen KR, Gilmour SJ, Zarka DG, Schabenberger O, Thomashow MF (1998) Arabidopsis CBF1 overexpression induces COR genes and enhances freezing tolerance. Science 280:104–106. 10.1126/science.280.5360.104 9525853 10.1126/science.280.5360.104

[CR50] Jørgensen M, Torp T, Mølmann JAB (2020) Impact of waterlogging and temperature on autumn growth, hardening and freezing tolerance of timothy (*Phleum pratense*). J Agron Crop Sci 206:242–251. 10.1111/jac.12385

[CR51] Jørgensen M (2017) Overvintring og vinterskader i eng. Available at: https://nibio.brage.unit.no/nibio-xmlui/handle/11250/2478939. Accessed 8 Apr 2024

[CR52] Kim SL, Lee S, Kim HJ, Nam HG, An G (2007) OsMADS51 is a short-day flowering promoter that functions upstream of Ehd1, OsMADS14, and Hd3a. Plant Physiol 145:1484–1494. 10.1104/pp.107.103291 17951465 10.1104/pp.107.103291 PMC2151696

[CR53] Klaus B, Strimmer K (2006) Fdrtool: estimation of (local) false discovery rates and higher criticism. 1.2.17. 10.32614/CRAN.package.fdrtool

[CR54] Kuparinen A, Savolainen O, Schurr FM (2010) Increased mortality can promote evolutionary adaptation of forest trees to climate change. For Ecol Manage 259:1003–1008. 10.1016/j.foreco.2009.12.006

[CR55] Lawrence M, Huber W, Pagès H, Aboyoun P, Carlson M, Gentleman R et al (2013) Software for computing and annotating genomic ranges. PLoS Comput Biol 9:e1003118. 10.1371/journal.pcbi.1003118 23950696 10.1371/journal.pcbi.1003118 PMC3738458

[CR56] Lenoir J, Gégout JC, Marquet PA, de Ruffray P, Brisse H (2008) A significant upward shift in plant species optimum elevation during the 20th century. Science 320:1768–1771. 10.1126/science.1156831 18583610 10.1126/science.1156831

[CR57] Lenth RV (2017) Emmeans: estimated marginal means, aka least-squares means. 1.10.4. 10.32614/CRAN.package.emmeans

[CR58] Li C, Liu X-J, Yan Y, Alam MS, Liu Z, Yang Z-K et al (2022) *OsLHY* is involved in regulating flowering through the *Hd1*- and *Ehd1*- mediated pathways in rice (*Oryza sativa* L.). Plant Sci 315:111145. 10.1016/j.plantsci.2021.111145 35067308 10.1016/j.plantsci.2021.111145

[CR59] Li H (2013) Aligning sequence reads, clone sequences and assembly contigs with BWA-MEM. 10.48550/ARXIV.1303.3997

[CR60] Liao Y, Smyth GK, Shi W (2014) FeatureCounts: an efficient general purpose program for assigning sequence reads to genomic features. Bioinformatics 30:923–930. 10.1093/bioinformatics/btt656 24227677 10.1093/bioinformatics/btt656

[CR61] Liu Z, Jia Y, Ding Y, Shi Y, Li Z, Guo Y et al (2017) Plasma membrane CRPK1-mediated phosphorylation of 14–3-3 proteins induces their nuclear import to fine-tune CBF signaling during cold response. Mol Cell 66:117-128.e5. 10.1016/j.molcel.2017.02.016 28344081 10.1016/j.molcel.2017.02.016

[CR62] Liu Y, Dang P, Liu L, He C (2019) Cold acclimation by the CBF–COR pathway in a changing climate: lessons from *Arabidopsis thaliana*. Plant Cell Rep 38:511–519. 10.1007/s00299-019-02376-3 30652229 10.1007/s00299-019-02376-3 PMC6488690

[CR63] Locato V, De Gara L (2018) Programmed cell death in plants: an overview. In: De Gara L, Locato V (eds) Plant programmed cell death: methods and protocols. Springer, New York, NY, pp 1–8. 10.1007/978-1-4939-7668-3_1 10.1007/978-1-4939-7668-3_1 29332281

[CR64] Love MI, Huber W, Anders S (2014) Moderated estimation of fold change and dispersion for RNA-seq data with DESeq2. Genome Biol 15:550. 10.1186/s13059-014-0550-8 25516281 10.1186/s13059-014-0550-8 PMC4302049

[CR65] MacKinnon KJ-M, Cole BJ, Yu C, Coomey JH, Hartwick NT, Remigereau M-S et al (2020) Changes in ambient temperature are the prevailing cue in determining *Brachypodium distachyon* diurnal gene regulation. New Phytol 227:1709–1724. 10.1111/nph.16507 32112414 10.1111/nph.16507

[CR66] Mitchell N, Whitney KD (2018) Can plants evolve to meet a changing climate? The potential of field experimental evolution studies. American J of Botany 105:1613–1616. 10.1002/ajb2.1170 10.1002/ajb2.1170 30281786

[CR67] Moran EV (2020) Simulating the effects of local adaptation and life history on the ability of plants to track climate shifts. AoB PLANTS 12:plaa008. 10.1093/aobpla/plaa008 32128105 10.1093/aobpla/plaa008 PMC7046178

[CR68] Nevo E, Fu Y-B, Pavlicek T, Khalifa S, Tavasi M, Beiles A (2012) Evolution of wild cereals during 28 years of global warming in Israel. Proc Natl Acad Sci U S A 109:3412–3415. 10.1073/pnas.1121411109 22334646 10.1073/pnas.1121411109 PMC3295258

[CR69] Nicotra AB, Atkin OK, Bonser SP, Davidson AM, Finnegan EJ, Mathesius U et al (2010) Plant phenotypic plasticity in a changing climate. Trends Plant Sci 15:684–692. 10.1016/j.tplants.2010.09.008 20970368 10.1016/j.tplants.2010.09.008

[CR70] Oostra V, Saastamoinen M, Zwaan BJ, Wheat CW (2018) Strong phenotypic plasticity limits potential for evolutionary responses to climate change. Nat Commun 9:1005. 10.1038/s41467-018-03384-9 29520061 10.1038/s41467-018-03384-9 PMC5843647

[CR71] Overland J, Wang M, Walsh J, Christensen J, Kattsov V, Chapman W (2011) Climate model projections for the Arctic. In: Snow, water, ice and permafrost in the arctic (SWIPA): climate change and the cryosphere, (AMAP), chapter 3–7

[CR72] Pandey GK, Grant JJ, Cheong YH, Kim BG, Li L, Luan S (2005) ABR1, an apetala2-domain transcription factor that functions as a repressor of ABA response in *Arabidopsis*. Plant Physiol 139:1185. 10.1104/pp.105.066324 16227468 10.1104/pp.105.066324 PMC1283757

[CR73] Pertea G, Pertea M (2020) GFF utilities: GffRead and GffCompare. F1000Res 9:ISCB Comm J-304. 10.12688/f1000research.23297.2 32489650 10.12688/f1000research.23297.1 PMC7222033

[CR74] Pin PA, Nilsson O (2012) The multifaceted roles of flowering locus t in plant development. Plant Cell Environ 35:1742–1755. 10.1111/j.1365-3040.2012.02558.x 22697796 10.1111/j.1365-3040.2012.02558.x

[CR75] Post E, Alley RB, Christensen TR, Macias-Fauria M, Forbes BC, Gooseff MN et al (2019) The polar regions in a 2 °C warmer world. Sci Adv 5:eaaw9883. 10.1126/sciadv.aaw9883 31840060 10.1126/sciadv.aaw9883 PMC6892626

[CR76] R Core Team (2022) *R*: a language and environment for statistical computing. *R* Foundation for statistical computing, Vienna, Austria. Available at: https://www.r-project.org/. Accessed 27 Jun 2022

[CR77] Rapacz M, Ergon Å, Höglind M, Jørgensen M, Jurczyk B, Østrem L et al (2014) Overwintering of herbaceous plants in a changing climate. Still more questions than answers. Plant Sci 225:34–44. 10.1016/j.plantsci.2014.05.009 25017157 10.1016/j.plantsci.2014.05.009

[CR78] Rauschkolb R, Li Z, Godefroid S, Dixon L, Durka W, Májeková M et al (2022) Evolution of plant drought strategies and herbivore tolerance after two decades of climate change. New Phytol 235:773–785. 10.1111/nph.18125 35357713 10.1111/nph.18125

[CR79] Robinson MD, McCarthy DJ, Smyth GK (2010) edgeR: a Bioconductor package for differential expression analysis of digital gene expression data. Bioinformatics 26:139–140. 10.1093/bioinformatics/btp616 19910308 10.1093/bioinformatics/btp616 PMC2796818

[CR80] Rognli OA (2013) Breeding for improved winter survival in forage grasses. In: Imai R, Yoshida M, Matsumoto N (eds) Plant and microbe adaptations to cold in a changing world. Springer New York, New York, NY, pp 197–208. 10.1007/978-1-4614-8253-6_17

[CR81] Sandve SR, Kosmala A, Rudi H, Fjellheim S, Rapacz M, Yamada T et al (2011) Molecular mechanisms underlying frost tolerance in perennial grasses adapted to cold climates. Plant Sci 180:69–77. 10.1016/j.plantsci.2010.07.011 21421349 10.1016/j.plantsci.2010.07.011

[CR82] Seo PJ, Mas P (2015) STRESSing the role of the plant circadian clock. Trends Plant Sci 20:230–237. 10.1016/j.tplants.2015.01.001 25631123 10.1016/j.tplants.2015.01.001

[CR83] Statistisk sentralbyrå (2025) SSB. Available at: https://www.ssb.no/en/jord-skog-jakt-og-fiskeri/jordbruk/statistikk/gardsbruk-jordbruksareal-og-husdyr. Accessed 6 Jan 2026

[CR84] Steinshamn H, Nesheim L, Bakken AK (2016) Grassland production in Norway. Grassl Sci Eur 21:101

[CR85] Stollewerk A, Kratina P, Sentis A, Chaparro-Pedraza C, Decaestecker E, De Meester L et al (2025) Plasticity in climate change responses. Biol Rev 100:2508–2527. 10.1111/brv.70056 40698780 10.1111/brv.70056 PMC12586308

[CR86] Sun X, Liu D, Zhang X, Li W, Liu H, Hong W et al (2013) SLAF-seq: an efficient method of large-scale de novo SNP discovery and genotyping using high-throughput sequencing. PLoS ONE 8:e58700. 10.1371/journal.pone.0058700 23527008 10.1371/journal.pone.0058700 PMC3602454

[CR87] Thivierge M-N, Bélanger G, Jégo G, Delmotte S, Rotz CA, Charbonneau É (2023) Perennial forages in cold-humid areas: adaptation and resilience-building strategies toward climate change. Agron J 115:1519–1542. 10.1002/agj2.21354

[CR88] Thorsen SM, Höglind M (2010) Assessing winter survival of forage grasses in Norway under future climate scenarios by simulating potential frost tolerance in combination with simple agroclimatic indices. Agric for Meteorol 150:1272–1282. 10.1016/j.agrformet.2010.05.010

[CR89] Uleberg E, Hanssen-Bauer I, van Oort B, Dalmannsdottir S (2014) Impact of climate change on agriculture in Northern Norway and potential strategies for adaptation. Clim Change 122:27–39. 10.1007/s10584-013-0983-1

[CR90] Wood DE, Lu J, Langmead B (2019) Improved metagenomic analysis with Kraken 2. Genome Biol 20:257. 10.1186/s13059-019-1891-0 31779668 10.1186/s13059-019-1891-0 PMC6883579

[CR91] Wu T, Hu E, Xu S, Chen M, Guo P, Dai Z et al (2021) clusterProfiler 4.0: a universal enrichment tool for interpreting omics data. Innovation New York N Y 2:100141. 10.1016/j.xinn.2021.100141 10.1016/j.xinn.2021.100141 PMC8454663 34557778

[CR92] Wuriyanghan H, Zhang B, Cao W-H, Ma B, Lei G, Liu Y-F et al (2009) The ethylene receptor ETR2 delays floral transition and affects starch accumulation in rice. Plant Cell 21:1473. 10.1105/tpc.108.065391 19417056 10.1105/tpc.108.065391 PMC2700534

[CR93] Yamamichi M, Hairston NG, Rees M, Ellner SP (2019) Rapid evolution with generation overlap: the double-edged effect of dormancy. Theor Ecol 12:179–195. 10.1007/s12080-019-0414-7

[CR94] Yang C, Hu H, Ren H, Kong Y, Lin H, Guo J et al (2016) LIGHT-INDUCED RICE1 regulates light-dependent attachment of leaf-type ferredoxin-NADP+ oxidoreductase to the thylakoid membrane in rice and *Arabidopsis*. Plant Cell 28:712–728. 10.1105/tpc.15.01027 26941088 10.1105/tpc.15.01027 PMC4826015

[CR95] Yang G, Chen T, Fan T, Lin X, Cui Y, Dong W et al (2024) Cathepsin B degrades RbcL during freezing-induced programmed cell death in *Arabidopsis*. Plant Cell Rep 43:81. 10.1007/s00299-023-03099-2 38418607 10.1007/s00299-023-03099-2

[CR96] Yu Y, Yang D, Zhou S, Gu J, Wang F, Dong J et al (2017) The ethylene response factor OsERF109 negatively affects ethylene biosynthesis and drought tolerance in rice. Protoplasma 254:401–408. 10.1007/s00709-016-0960-4 27040682 10.1007/s00709-016-0960-4

[CR97] Zhou X, Liu Q, Xie F, Wen C-K (2007) RTE1 is a Golgi-associated and ETR1-dependent negative regulator of ethylene responses. Plant Physiol 145:75–86. 10.1104/pp.107.104299 17644624 10.1104/pp.107.104299 PMC1976582

