# Supplementary material for: Allele frequency and gene expression differences under key winter stresses in temporal populations of two timothy cultivars
Source: Theor Appl Genet. 2026 May 23;139(6):161. doi: 10.1007/s00122-026-05270-1 (PMC13198515; doi:10.1007/s00122-026-05270-1)
Supplement: Supplementary file 1 — Supplementary file1 (DOCX 1197 KB) [file 122_2026_5270_MOESM1_ESM.docx]

## Supplementary Figures


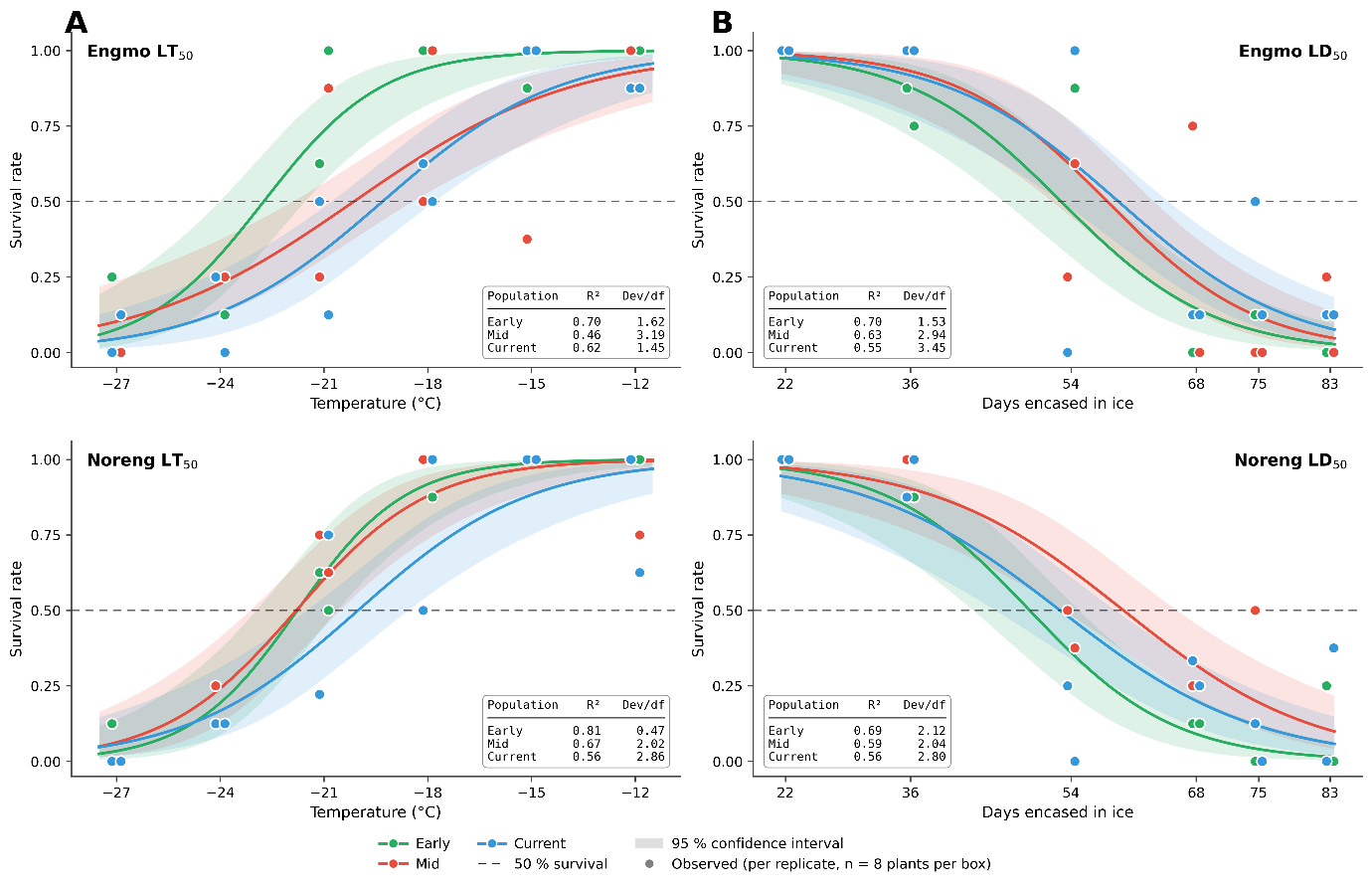


**Figure S1:** A) Estimated LT_50_ and B) LD_50_, of the temporal populations. Coloured points are the observed survival proportions of individual freezer boxes (n = 8 plants per box, two boxes per cultivar × population × condition. Solid lines are the fitted logistic curves from per-population binomial GLMs (predictor: temperature in °C for panels A, or days under ice for panels B). Coloured shaded bands are the corresponding 95 % confidence intervals of the predicted survival probability. The dashed horizontal line marks the 50 % survival level used to define LT₅₀ and LD₅₀. The inset table in each panel reports two goodness-of-fit statistics for the three per-population GLMs: McFadden's pseudo-R², the proportion of the null-model log-likelihood explained by the fitted model (higher is better), and Dev/df, the ratio of residual deviance to residual degrees of freedom (values close to 1 indicate adequate binomial fit).


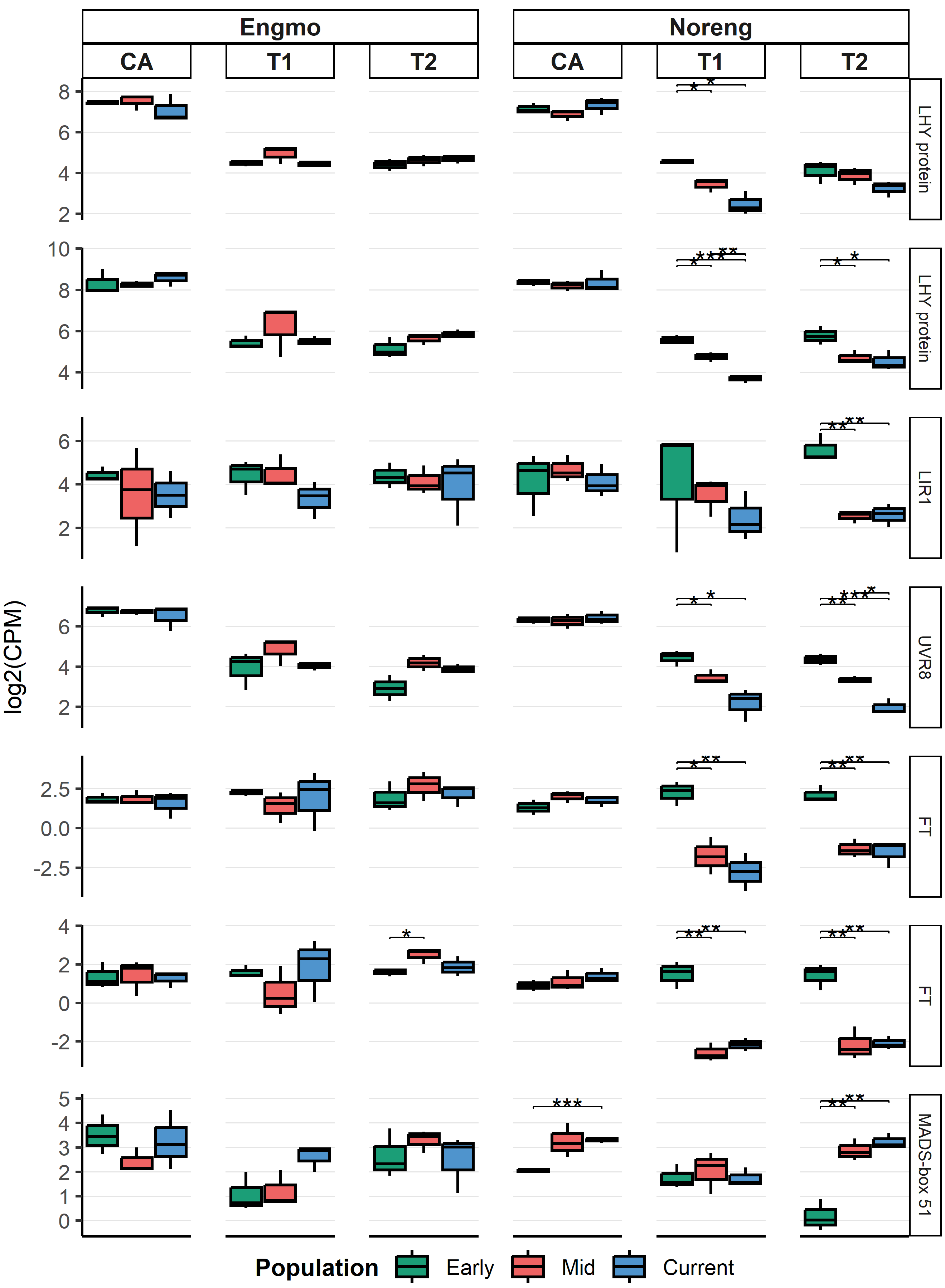


**Figure S2:** DEGs between populations of Noreng under freezing stress that are linked to photoperiod and flowering. The statistical significance is based on a t-test between populations at a given treatment.


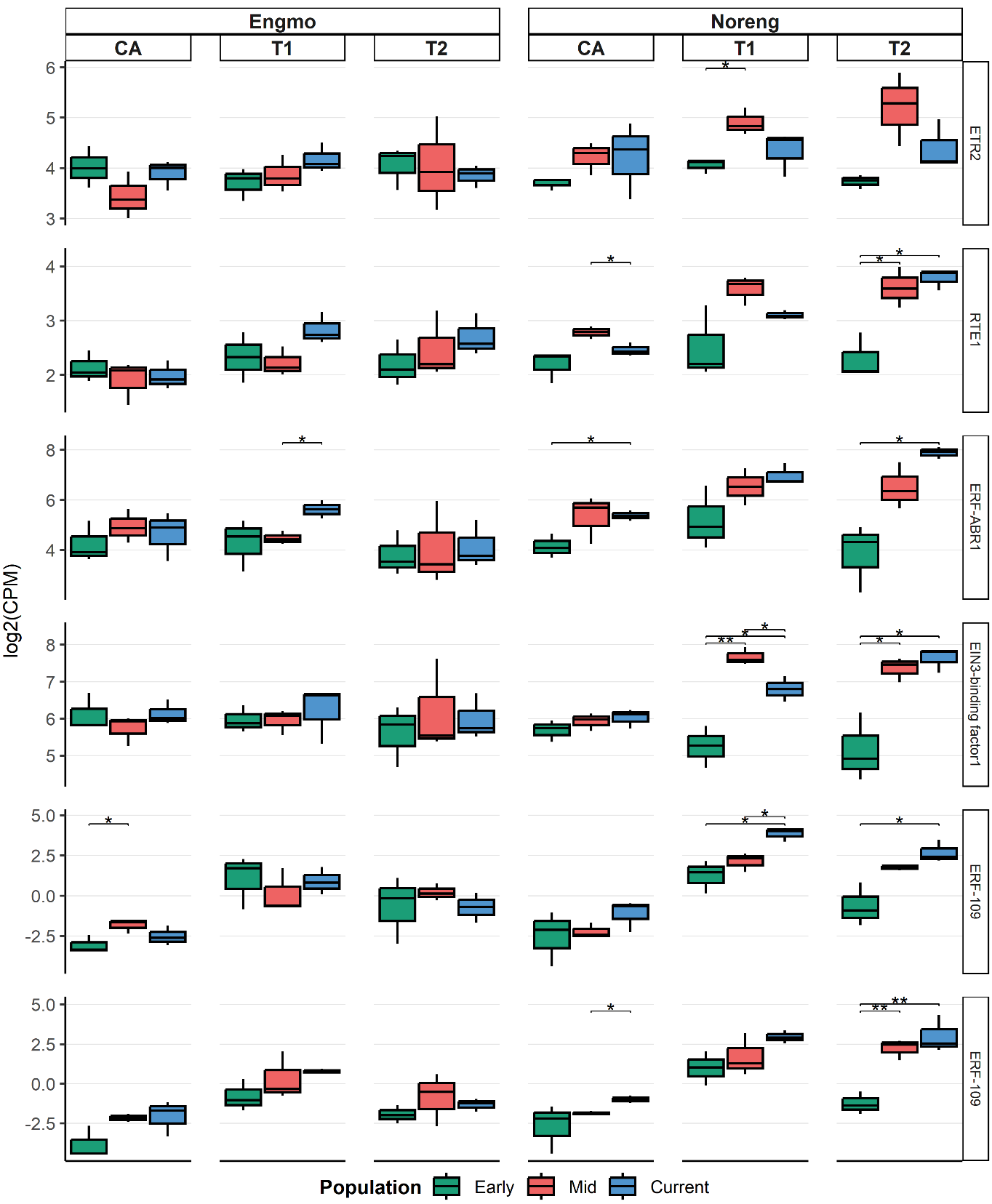


**Figure S3:** DEGs between populations of Noreng under freezing stress that are linked ethylene- and abscisic acid-mediated transcriptional regulation. The statistical significance is based on a t-test between populations.


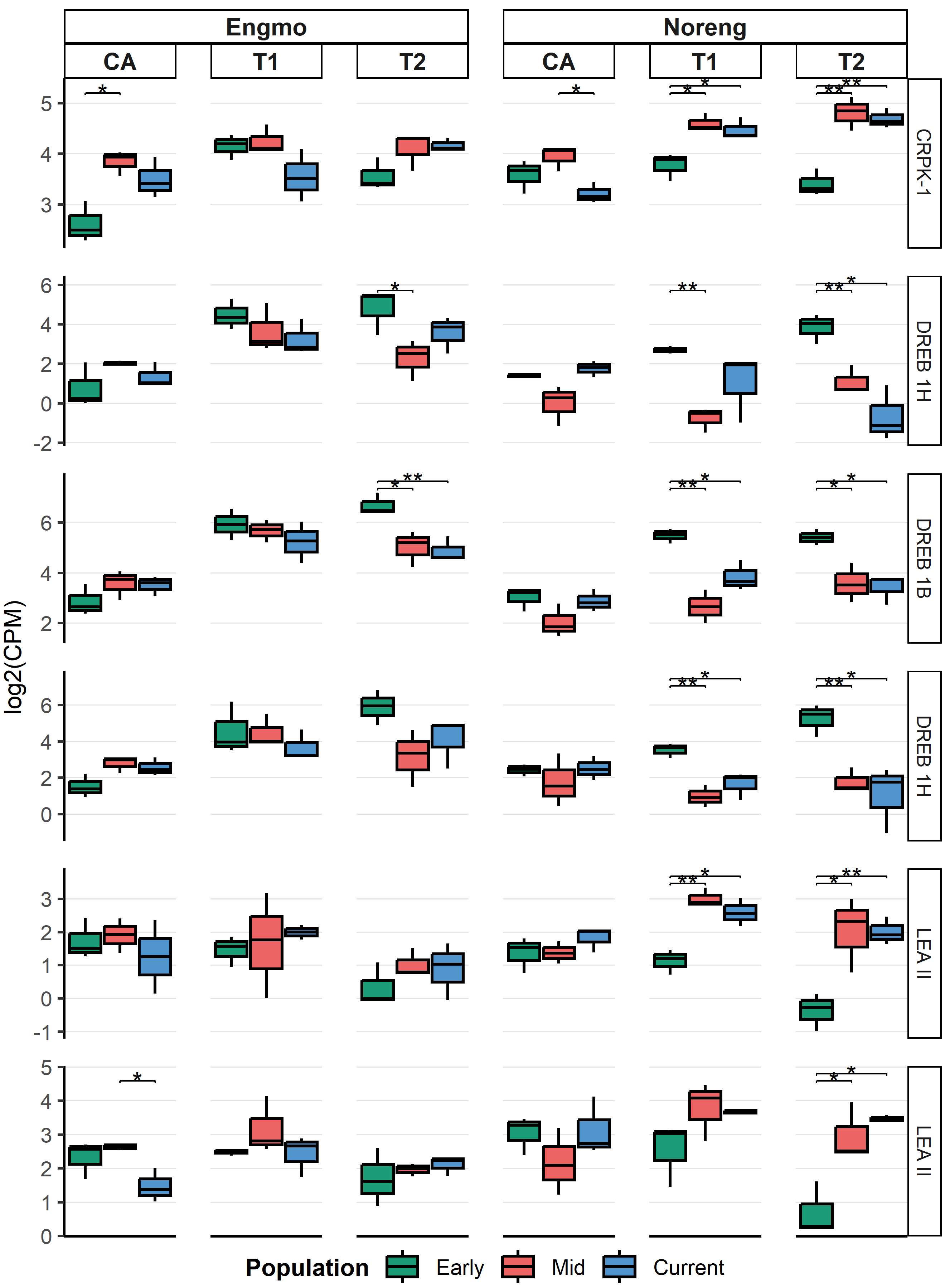


**Figure S4:** DEGs between populations of Noreng under freezing stress that are linked to freezing stress responses. The statistical significance is based on a t-test between populations.


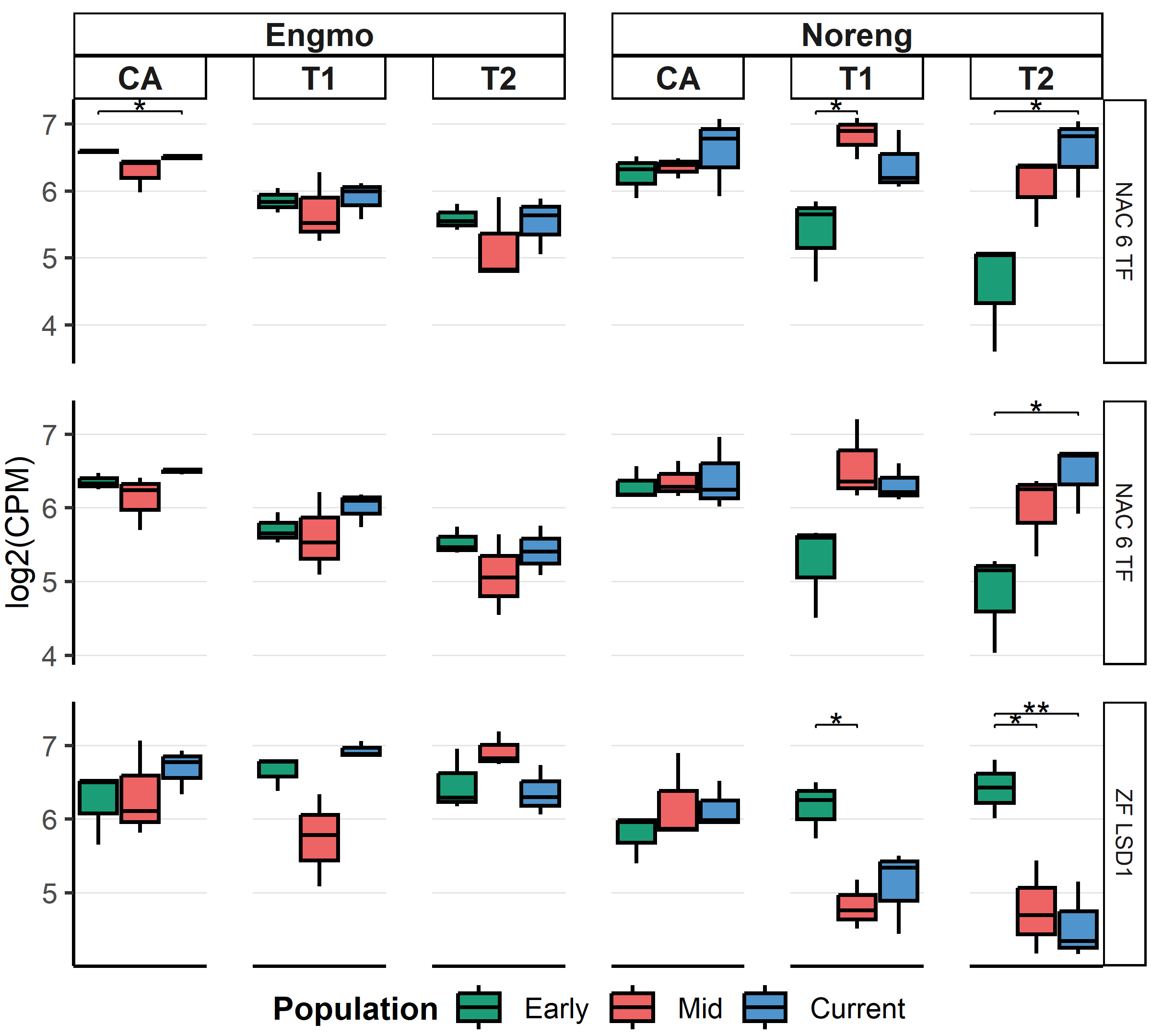


**Figure S5:** DEGs between populations of Noreng under freezing stress that are linked to programmed cell death. The statistical significance is based on a t-test between populations.


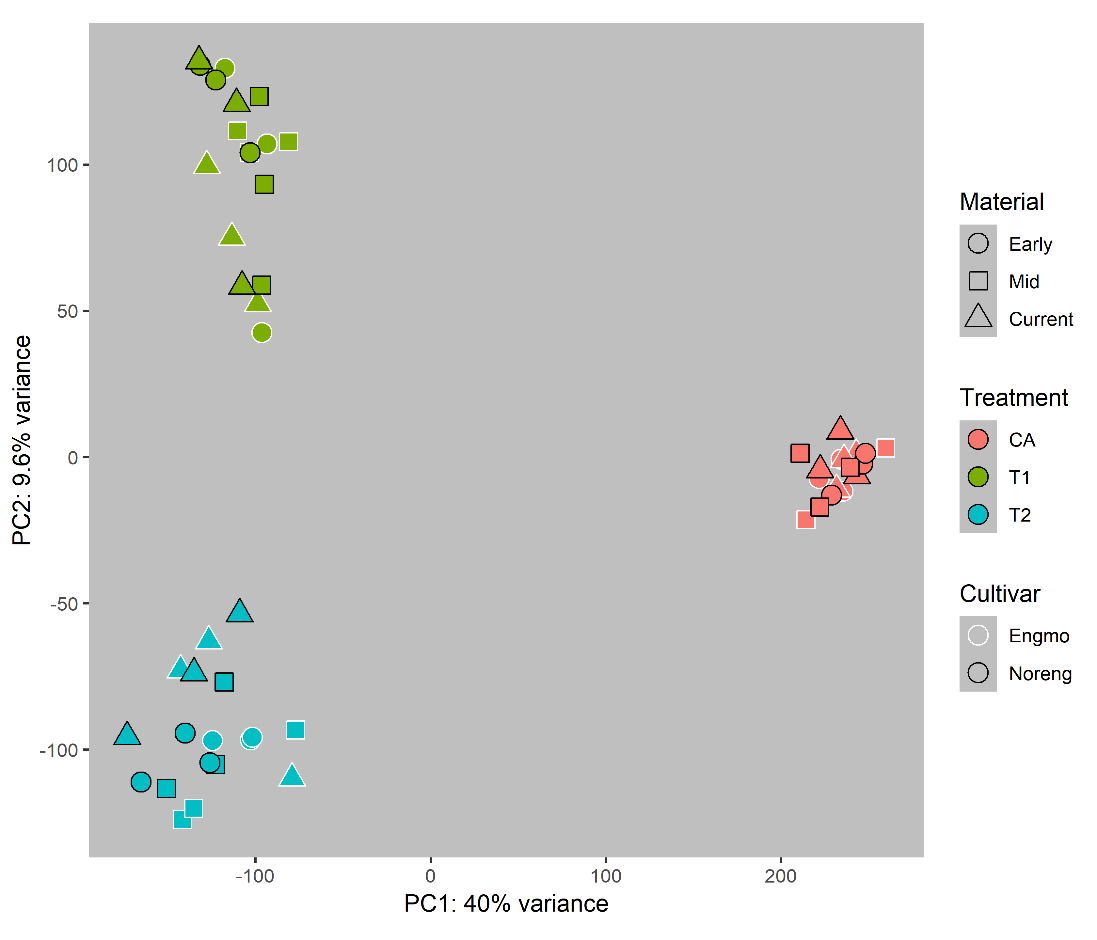


**Figure S6:** PCA plot based on gene expression under ice encasement stress. CA=cold acclimation, T1=36 days (< LD_50_), T2=68 days (> LD_50_). Early=1988-98, mid=2003-10, current=2020.


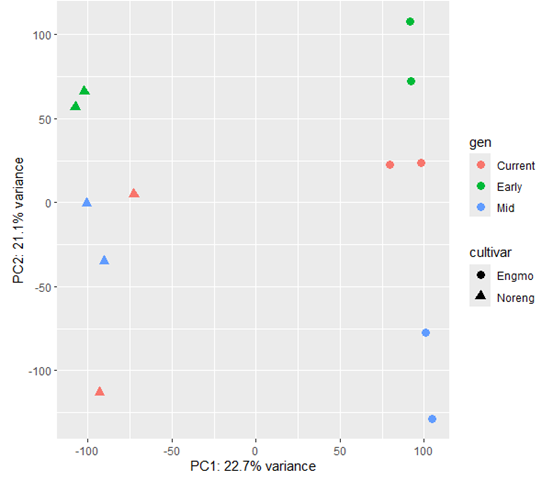


**Figure S7:** PCA plot based on allele frequencies at the replicate level.
